# Active and social life is associated with lower non-social fearfulness in pet dogs

**DOI:** 10.1038/s41598-020-70722-7

**Published:** 2020-08-13

**Authors:** Emma Hakanen, Salla Mikkola, Milla Salonen, Jenni Puurunen, Sini Sulkama, César Araujo, Hannes Lohi

**Affiliations:** 1 Department of Veterinary Biosciences, University of Helsinki, Helsinki, Finland; 2 Department of Medical and Clinical Genetics, University of Helsinki, Helsinki, Finland; 3 Folkhälsan Research Center, Helsinki, Finland

**Keywords:** Behavioural ecology, Ecological epidemiology, Animal behaviour, Epidemiology

## Abstract

Behavioural problems are leading welfare issues in domestic dogs. In particular, anxiety-related behavioural problems, such as fearfulness and noise sensitivity are highly prevalent conditions that cause distress to fearful dogs. To better understand the environmental factors associated with non-social fear, including noise sensitivity, fear of novel situations, and fear of surfaces and heights, a large online survey including data on 13,700 Finnish pet dogs was performed by the dog owners. After fulfilling inclusion criteria, this data consisted of 9,613 dogs with fear of fireworks, 9,513 dogs with fear of thunder, 6,945 dogs with fear of novel situations, and 2,932 dogs with fear of surfaces and heights. Logistic regression analyses revealed that dogs with frequent non-social fear had experienced less socialisation during puppyhood, were more often neutered, had inexperienced owners, lived without conspecifics, participated less frequently in activities or training, and lived in more urban environments. In addition, we identified several breed differences, and a tendency of more common non-social fear in small dog breeds, which suggests a genetic background. Non-social fearfulness has a negative effect on well-being of the dogs. Our findings suggest that the socialisation and the living environment and the value of other dogs’ company and owner interaction via activities and training may improve the well-being of the dogs.

## Introduction

Fear and anxiety-related behavioural problems, such as fear of new situations, fear towards humans, and noise sensitivity, are major and global welfare problems of pet dogs that often lead to relinquishment or euthanasia^1–5^. These behavioural problems have a negative effect on the wellbeing of both the dog and its owner, as owners are often less satisfied and are more weakly attached to dogs that show problematic behaviour^6–9^.

Fear and anxiety are used as terms to describe similar physiological signs of heightened arousal.

Fear is a normal emotion that is found in all animal species. It is transient state elicited through threatening stimuli such as noise or smell^10–13^. Anxiety can be described as the anticipation of a perceived threat and may be displayed without threatening stimulus^12,13^.

Fear can be classified into two categories based on the stimuli that causes fear. Social fear includes fear of other dogs or unfamiliar people, whereas non-social fear includes fear of different objects, loud noises and novel situations^14^. The dog can show different fearful signs depending on the stimuli. For instance, dogs that are afraid of strangers usually avoid or withdraw, bark, or have a low tail position. Dogs that are fearful of novel situations usually pant, express a desire to leave the situation, or stay close to their owners^15^.

Fearfulness is influenced by both genetic and environmental factors. Canine fearfulness is highly heritable^16–23^ and studies have already identified some candidate loci and genes that affect fearfulness^24–27^. Environmental factors discovered by previous studies include socialisation, training and daily exercise, owner’s previous dog experience, and company of conspecifics among other things^28–36^. Fearfulness also correlates with some physiological measures, as fearful dogs have reduced heart rate variability^37^ and differences in their metabolism compared to non-fearful dogs^38,39^.

Non-social fear is a common problem in dogs. As shown in our recent study from Finnish pet dogs, the prevalence of noise sensitivity, fear of novel situations, and fear of surfaces and heights were 32%, 11%, and 24%, respectively^40^. As extreme non-social fear can hinder the life of a dog, more research is needed to better understand the associated environmental factors to improve the prevention and management of these fear-related problems. This is particularly important with the fear of surfaces and high places as, to best of our knowledge, no previous studies on environmental factors exist. Identification of the most important factors could possibly enable the prevention and management of fear-related problems. Thus, our objective in this study was to explore the environmental and demographic factors associated with non-social fearfulness.

## Results

### Demographics

We studied the environmental factors for fear of fireworks (n = 9,613), thunder (n = 9,513), novel situations (n = 6,945), and fear of heights and surfaces (n = 2,932). The fear of fireworks sample included 6,732 non-fearful and 2,881 fearful dogs with a mean age of 4.8 years (range 2 months to 18 years).

The fear or thunder sample included 7,809 non-fearful and 1,704 fearful dogs with a mean age of 4.7 years (range 2 months to 17 years). The fear of novel situations sample included 6,062 non-fearful and 883 fearful dogs with a mean age of 4.6 years (range 2 months to 18 years). The fear of surfaces and heights sample included 1,212 non-fearful and 1,720 fearful dogs with a mean age of 5.1 years (range 3 months to 16 years). In all sample sets, 52% of the dogs were females. More detailed descriptive statistics and lists of included breeds and the number of individuals per breed are presented in the Supplementary Table S1.

### Factors associated with fear of fireworks

Logistic regression analysis identified several environmental and demographic factors associated with fear of fireworks, including age, socialisation score, neutering, activities/training, breed, owner’s dog experience, and dogs in the family (Table 1).

**Table 1:** Associations between the demographic and environmental variables with fear of fireworks and fear of thunder of the final models in the logistic regression analyses.

Variable	Fear of fireworks			Fear of thunder		
	DF	χ^2^	p*-*value	DF	χ^2^	p*-*value
Age	1	146.8006	< 0.0001*	1	187.6205	< 0.0001*
Age^2^	1	73.9781	< 0.0001*	1	92.5000	< 0.0001*
Sex	1	0.0188	0.8908*	1	0.4848	0.4863*
Sterilisation	1	29.0604	< **0.0001**	1	43.2807	< **0.0001**
Socialisation score	1	26.1462	< **0.0001**	1	21.7946	< **0.0001**
Activities/training	2	32.4767	< **0.0001**	2	18.8182	**0.0006**
Breed	23	241.4084	< **0.0001**	22	172.2286	< **0.0001**
Owner’s dog experience	1	62.3541	< 0.0001*			
Other dogs in family	1	46.9982	< **0.0001**	1	35.3653	< **0.0001**
Daily exercise				3	7.0714	0.1388
Body size				2	12.6924	0.0018*

p*-*values are controlled for false discovery rate except for a priori contrasts. Significant values are in bold (p-value < 0.05) and a priori effects are denoted with*. N = 9,613 (fear of fireworks), n = 9,513 (fear of thunder).

**Figure 1:**
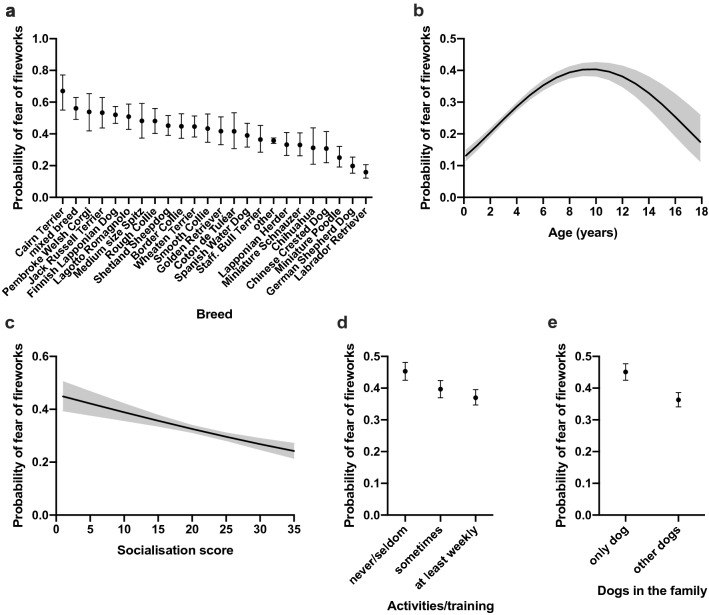
Logistic regression analyses on the effects of breed (**a**), age (**b**), socialisation (**c**), activities/training (**d**), and dogs in the family (**e**) for the fear of fireworks. The Y axis shows the predicted probability of belonging to the "high" fear group. Error bars (**a**, **d**, **e**) and grey lines (**b**, **c**) indicate the 95% confidence limits*.* n = 9,613.

We found breed differences in the likelihood of fear of fireworks. The most fearful breeds were Cairn Terrier, mixed breed, and Pembroke Welsh Corgi: the least fearful breeds were Labrador Retriever, German Shepherd Dog, and Miniature Poodle (Fig. 1a). All pairwise breed differences are shown in Supplementary Table S8.

The probability of fear of fireworks increased with age until 10 years of age and decreased thereafter (χ^2^ = 146.8, DF = 1, p < 0.0001, quadratic effect: χ^2^ = 73.98, DF = 1, p < 0.0001) (Table 1, Fig. 1b). Dogs with less socialisation experiences had a higher probability of fear of fireworks (χ^2^ = 26.15, DF = 1, p < 0.0001) (Table 1, Fig. 1c).

Dogs participating less frequently in activities and training were more fearful towards fireworks. More specifically, dogs participating in activities and training only seldom/never were more likely to be fearful than dogs that trained sometimes (odds ratio [OR] = 1.26, p = 0.0031) or at least weekly (OR = 1.41, p = 0.0004) (Supplementary Table S2, Fig. 1d). If the dog was the only dog in the family, it had a higher probability to show fear of fireworks than if the dog had dog company (OR = 1.44, p = 0.0004) (Supplementary Table S2, Fig. 1e).

There was no significant difference between female and male dogs (OR = 1.01, p = 0.8908) (Supplementary Table S2, Supplementary Fig. S1a). Neutered dogs were more likely to show fear of fireworks than intact dogs (OR = 0.75, p = 0.0004) (Supplementary Table S2, Supplementary Fig. S1b). If the dog was the owner’s first dog, it was more likely fearful than if the dog was not the first dog (OR = 1.53, p < 0.0001) (Supplementary Table S2, Supplementary Fig. S1c).

### Factors associated with fear of thunder

Logistic regression analysis identified several environmental and demographic factors associated with fear of thunder, including age, socialisation score, neutering, activities/training, breed, dogs in the family, daily exercise, and body size (Table 1).

**Figure 2:**
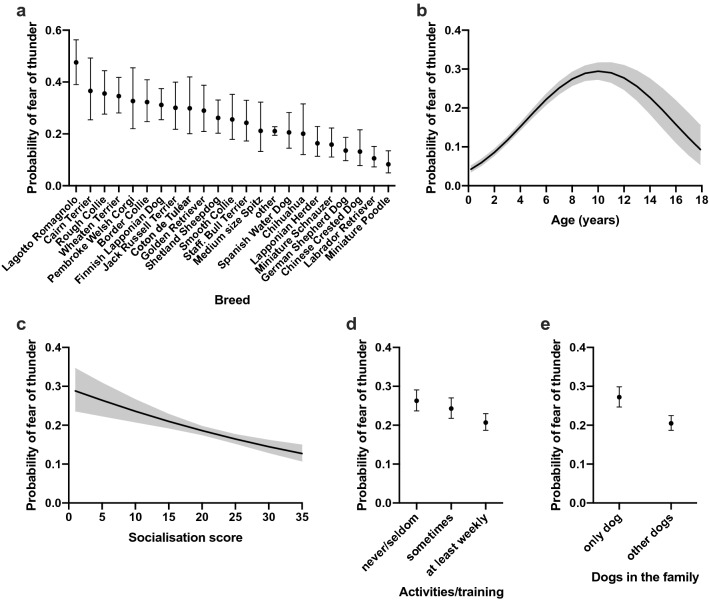
Logistic regression analyses on the effects of breed (**a**), age (**b**), socialisation (**c**), activities/training (**d**), and dogs in the family (**e**) for the fear of thunder. The Y axis shows the predicted probability of belonging to the "high" fear group. Error bars (**a**, **d**, **e**) and grey lines (**b**, **c**) indicate the 95% confidence limits. n = 9,513.

There were also differences between several breeds in the likelihood of fear of thunder. Lagotto Romagnolo, Cairn Terrier, and Rough Collie were the most fearful breeds whereas Miniature Poodle, Chinese Crested Dog, and Labrador Retriever were the least fearful breeds (Fig. 2a). All pairwise breed differences are shown in Supplementary Table S9.

The probability of fear of thunder increased with age until 10 years of age and decreased thereafter (χ^2^ = 187.62, DF = 1, p < 0.0001, quadratic effect: χ^2^ = 92.5, DF = 1, p < 0.0001) (Table 1, Fig. 2b). Dogs with less socialisation experiences were more likely fearful towards thunder (χ^2^ = 21.79, DF = 1, p < 0.0001) (Table 1, Fig. 2c).

Dogs participating less frequently in activities and training were more fearful towards thunder. More specifically, dogs participating in activities and training only seldom/never or sometimes were more likely fearful than dogs that trained at least weekly (OR = 1.36, *P* = 0.0001; OR = 1.23, p = 0.0037, respectively) (Supplementary Table S3, Fig. 2d). If the dog was the only dog in the family, it had a higher probability to show fear of thunder than if the dog had dog company (OR = 1.45, p = 0.0006) (Supplementary Table S3, Fig. 2e).

Female and male dogs were equally fearful of thunder (OR = 0.96, p = 0.4863) (Supplementary Table S3, Supplementary Fig. 2a). Dogs that exercised 1–2 h or 2–3 h were less fearful toward thunder than dogs that exercised more than 3 h (OR = 0.83, *P* = 0.0412; OR = 0.78, p = 0.008, respectively) (Supplementary Table S3, Supplementary Fig. S2b). Neutered dogs were more likely to have fear of thunder than intact dogs (OR = 1.55, p = 0.0006) (Supplementary Table S3, Supplementary Fig. S2c). While large dogs were less likely to have fear of thunder than to small dogs (OR = 0.72, p = 0.0004), but there was no significant difference between small and medium dogs (OR = 0.82, p = 0.0981), or between medium and large dogs (OR = 0.88, p = 0.1669) (Supplementary Table S3, Supplementary Fig. S2d).

### Factors associated with fear of novel situations

Logistic regression analysis identified several environmental and demographic factors associated with fear of novel situations, including age, socialisation, urban environment score, sterilisation, family size, activities/training, and the interaction of sex and sterilisation (Table 2).

**Table 2:** Associations between the demographic and environmental variables with fear of novel situations and fear of surfaces and heights of the final models in the logistic regression analyses.

Variable	Fear of novel situations			Fear of surfaces and heights		
	DF	χ^2^	p*-*value	DF	χ^2^	p*-*value
Age	1	7.5182	0.0061*	1	1.7503	0.3616
Age^2^	1	12.6047	0.0004*	1	3.3830	0.2569
Sex	1	5.1035	0.0239*	1	0.0125	0.9111*
Sterilisation	1	36.4091	< **0.0001**			
Sex*sterilisation	1	6.8662	**0.0157**			
Socialisation score	1	157.1755	< 0.0001*	1	0.0701	0.7913*
Socialisation score^2^	1			1	1.2829	0.2574
Activities/training	2	49.5731	< **0.0001**	2	50.4733	< **0.0001**
Urban environment score	1	12.3145	**0.0010**		7.2743	0.0070*
Family size	4	21.5506	**0.0006**			
Breed				22	66.5632	**0.0002**
Owner’s dog experience				2	14.5510	**0.0027**
Other dogs in family				1	40.6092	< **0.0001**
Daily exercise				3	10.8998	0.07619
Body size				2	55.0844	< 0.0001*
Fearfulness				2	95.5480	< 0.0001*

p*-*values are controlled for false discovery rate except for a priori contrasts. Significant values are in bold (p-value < 0.05) and a priori effects are denoted with*. n = 6,945 (fear of novel situations), n = 2,932 (fear of heights and surfaces).

**Figure 3:**
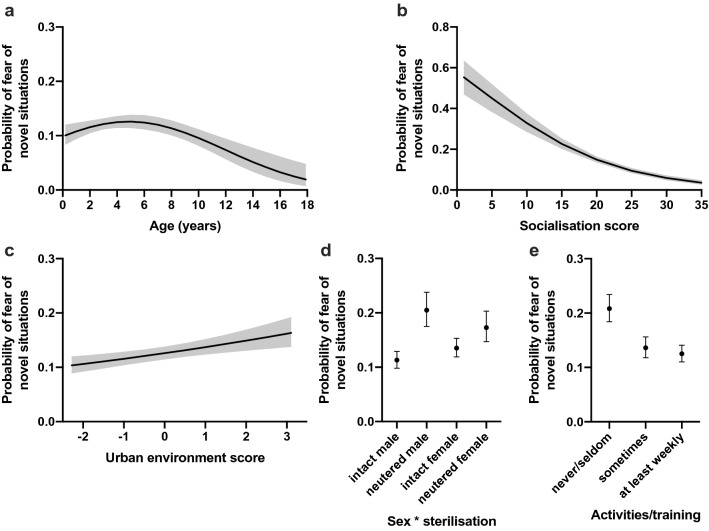
Logistic regression analyses on the effects of age (**a**), socialisation (**b**), urban environment (**c**), interaction between sex and sterilisation (**d**), and activities/training (**e**) for the fear of novel situations. The Y axis shows the predicted probability of belonging to the "high" fear group. Grey lines (**a**–**c**) and error bars (**d**, **e**) indicate the 95% confidence limits. n = 6,945.

The probability of fear of novel situations increased with age until 5 years of age and decreased thereafter (χ^2^ = 7.52, DF = 1, p = 0.0061, quadratic effect: χ^2^ = 12.6, DF = 1, p = 0.0004) (Table 2, Fig. 3a). Dogs with less socialisation experiences had higher probabilities of fear of novel situations (χ^2^ = 157.18, DF = 1, p < 0.0001) (Table 2, Fig. 3b). Dogs with higher “urban environment score” were more fearful in novel situations (χ^2^ = 12.31, DF = 1, p = 0.0010) (Table 2, Fig. 3c).

We found a significant interaction between sex and sterilisation. Intact males were less fearful in novel situations when compared to intact females (OR = 0.81, p = 0.0373). There was no difference between neutered males and females (OR = 1.23, p = 0.1292). Intact males were less fearful in novel situations than neutered males (OR = 0.494, p = 0.0003); and intact females were also less fearful when compared to neutered females (OR = 0.75, p = 0.0187) (Supplementary Table S4, Fig. 3d).

Dogs participating less frequently in activities and training were more fearful in novel situations. More specifically, dogs participating in activities and training only seldom/never were more likely fearful than dogs that trained sometimes (OR = 1.67, p = 0.0003) or at least weekly (OR = 1.84, p = 0.0003) (Supplementary Table S4, Fig. 3e).

A larger family size was associated with higher probabilities of fear of novel situations. Dogs living with one adult (“single”) were less likely to have fear of novel situations than dogs living in families with two children (OR = 0.66, p = 0.0048) or in a larger family (OR = 0.57, p = 0.0003). Dogs living with couples were less likely fearful than dogs living with a larger family (OR = 0.70, p = 0.0025) (Supplementary Table S4, Supplementary Fig. S3a).

### Factors associated with fear of surfaces and heights

Logistic regression analysis identified several environmental and demographic factors associated with fear of surfaces and heights, including age, socialisation, urban environment, sex, activities/training, breed, owner’s dog experience, dogs in the family, daily exercise, body size, and fearfulness (Table 2).

**Figure 4:**
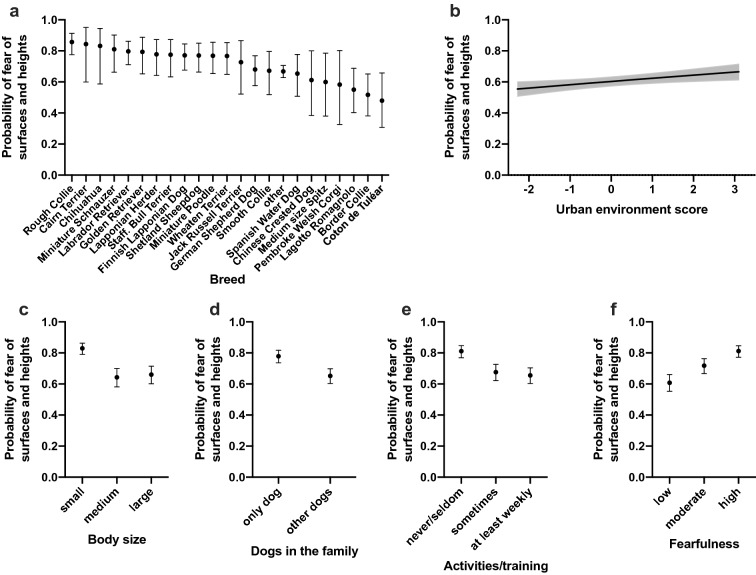
Logistic regression analyses on the effects of breed (**a**), urban environment (**b**), body size (**c**), dogs in the family (**d**), activities/training (**e**), and fearfulness (**f**) for the fear of surfaces and heights. The Y axis shows the predicted probability of belonging to the "high" fear group. Error bars (**a**, **c**, **d**, **e**, **f**) and grey line (**b**) indicate the 95% confidence limits. n = 2,932.

We found several breed differences in the likelihood of fear of surfaces and heights. Rough Collie, Cairn Terrier, and Chihuahua had the highest probabilities of fear of surfaces and heights whereas Coton de Tuléar, Border Collie, and Lagotto Romagnolo had the lowest probabilities of fear of surfaces and heights (Fig. 4a). All pairwise breed differences are shown in Supplementary Table S10.

Dogs living in a more urban environment had a higher probability of fear of surfaces and heights (χ^2^ = 7.27, DF = 1, p = 0.0007) (Table 2, Fig. 4b). Large and medium dogs were less likely to show fear of surfaces and heights than small dogs (OR = 0.40, p < 0.0001; OR = 0.369, p = 0.0021, respectively) (Supplementary Table S5, Fig. 4c). Only dogs in the families were more fearful than dogs living with other dogs (OR = 1.88, p = 0.0021) (Supplementary Table S5, Fig. 4d).

Dogs participating less frequently in activities and training were more likely to show fear of surfaces and heights. More specifically, dogs participating in activities and training only seldom/never were more likely fearful than dogs training sometimes (OR = 2.06, p = 0.0021) or at least weekly (OR = 2.26, p = 0.0021) (Supplementary Table S5, Fig. 4e).

Higher fearfulness was associated with higher probabilities of also having fear of surfaces and heights, as dogs showing no fear were less likely to have fear of surfaces and heights than dogs showing moderate (OR = 0.61, p < 0.0001) or high (OR = 0.36, p < 0.0001) levels of fear. Dogs with moderate fearfulness were also less likely to have a fear of surfaces and heights than dogs with high fearfulness (OR = 0.59, p < 0.0001) (Supplementary Table S5, Fig. 4f).

Age was not associated with fear of surfaces and heights (linear effect: χ^2^ = 1.75, DF = 1, p = 0.186; quadratic effect: χ^2^ = 3.38, DF = 1, p = 0.066) (Table 2, Supplementary Fig. S4a). Socialisation score (χ^2^ = 0.07, DF = 1, p = 0.712; quadratic effect: χ^2^ = 1.28, DF = 1, p = 0.257) (Table 2, Supplementary Fig. S4b) and daily exercise (χ^2^ = 10.90, DF = 3, p = 0.076) (Supplementary Table S5, Supplementary Fig. S4e) were also not associated. If the dog was the owner’s first dog, it was more likely to show fear of surfaces and heights than if the dog was not the first dog (OR = 1.49, p = 0.0021) (Supplementary Table S5, Supplementary Fig. S4c). There was also no significant difference between the female and male dogs (OR = 1.01, p = 0.911) (Supplementary Table S5, Supplementary Fig. S4d).

**Figure 5:**
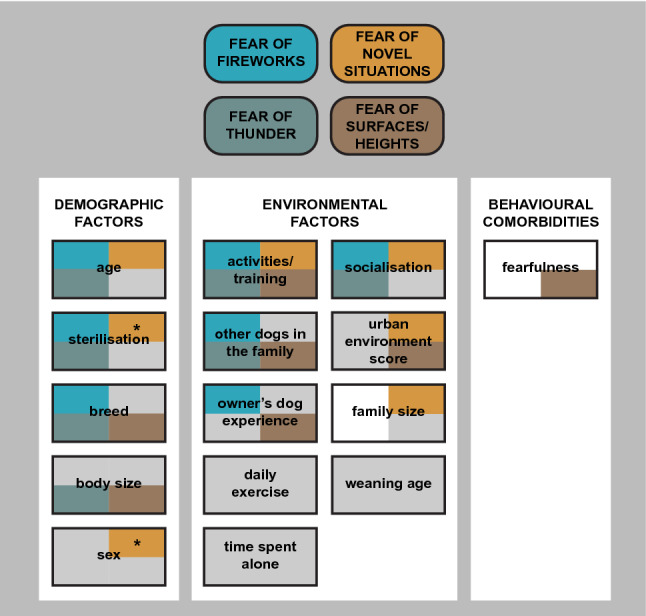
Demographic and environmental factors and behavioural comorbidities included in the logistic regression analyses of non-social fear subtraits. The subtraits have been assigned a colour and factors significant for each subtrait are marked with the same colour. White colour indicates that the factor was not tested in the analysis and grey colour indicates that the factor was not significant in the final model or was excluded in the model selection. The sex and sterilisation interaction was added in the model for the fear of novel situations, and it is denoted with asterisk.

A summary of demographic and environmental factors associated with non-social fear in dogs are shown in Fig. 5.

## Discussion

We have performed one of the largest survey studies in dog behaviour to understand the environmental and demographic factors that predispose to fear. We targeted here four subtraits of non-social fear, namely fear of fireworks, thunder, novel situations, and surfaces and heights, and revealed a diverse set of associated risk factors while also highlighting the role of genetic predisposition. This study emphasises the importance of early life socialisation and an active and social lifestyle to prevent non-social fear, provides the first description of factors related to fear of surfaces and heights, and identifies novel factors of non-social fear such as urbanisation. The insights gained from this study can be used to improve the management, living conditions, welfare, and breeding of pet dogs while guiding future molecular studies.

Our results suggest that non-social fear is more common in adulthood. The probability of noise sensitivity and fear of novel situations was higher for young dogs and lower for puppies and older dogs. Older dogs may suffer from hearing loss and may also be sufficiently habituated to novel stimuli or are not introduced anymore to new situations, which could possibly explain our non-linear results. Previous studies have reported similar findings with increased noise sensitivity^15,40–43^ and fear of novel situations by age^40^.

We replicated earlier studies as we found an association between sterilisation and non-social fear^34,35,44,45^. Noise sensitivity and fear of novel situations were more common in neutered dogs than in intact dogs. We also discovered a significant interaction between sex and sterilisation in fear of novel situations. Intact females were more fearful in novel situations than intact males, but no difference was found between neutered males and females. However, a recent review^46^ concluded that the effect of sterilisation on fearful behaviour is inconsistent and even contradictory. Thus, our results should be interpreted with caution.

We observed breed differences in non-social fear, suggesting genetic susceptibility. We identified breed differences in fear of fireworks, fear of thunder, and fear of surfaces and heights. The Cairn Terrier was one of the most fearful breeds in all three subtraits, whereas the Chinese Crested Dog was one of the least fearful breeds. Many breeds showed either high or low fearfulness in many traits, suggesting comorbidity in non-social fear. However, there was some breed variability. For instance, the Pembroke Welsh Corgi had one of the highest probabilities of fear of fireworks and thunder, but the lowest in fear of surfaces and heights, whereas the opposite was true for Lapponian Herder, Miniature Schnauzer, Chihuahua, and Labrador Retriever. Although breed differences have previously been found for non-social fear^15,33,40,43–45,47^, it is important to note that comparing previous studies may not be meaningful as the results are based on the breeds available for each specific study cohort. We also note that in our study some breeds had large confidence intervals due to small sample sizes. Thus, larger cohorts are required in future research.

In addition to breed differences, we found that small dogs were more fearful towards thunder and surfaces and heights than medium- or large-sized dogs. Previous studies have found correlation between body size and non-social fear, with smaller dogs showing more fearful reactions^48,49^. Interestingly, body size was not associated with fear of fireworks. A recent study suggested that behavioural problems are considered more harmful in larger dogs and thus are then selected against in breeding choices. On the other hand, the owners of small dogs may protect their dogs and do not let them go into potentially fearful situations^48^. It is possible that the owners of the small dogs might avoid firework displays more efficiently than the sound of a thunder, as the fireworks displays are planned and notified beforehand. Smaller dogs may feel threatened by heights, as heights (such as stairs), are taller compared to a small than to a large dog. Smaller dogs may also have more difficulties on different surfaces, as their small paws may make it difficult to walk properly, such as on slippery floors. Some surfaces, such as metal grids, can even be painful for smaller dogs. We note that smaller dogs are also easier to carry over difficult surfaces such as metal grids. It is also possible fearful behaviour and body size share genetic components^48^.

We also found a novel association between general fearfulness and fear of surfaces and heights: fearful dogs had a higher likelihood of fear of surfaces and heights. Interestingly, visual height intolerance and acrophobia (intense fear of heights) have high co-morbidity with anxiety and other mental disorders in humans. While the causality and reason for this is unknown, genetic distribution, environmental childhood factors, and personality characteristics have been suggested^50–52^. Our recent study using the same cohort of dogs also found high co-morbidity with fear of surfaces and heights and other behavioural problems^40^.

We found that dogs that did not actively (never or seldom) engage in activities and training showed more non-social fear than dogs that train at least weekly. The benefits of physical activity and mental health have been observed in humans^53,54^. Exercise increases serotonin levels, decreases stress, and works in the same way as selection serotonin reuptake inhibitors^55,56^. The same phenomenon that physical activity reduces stress could be seen in dogs. A recent study revealed that dogs with noise sensitivity exercised less than non-fearful dogs^34^. However, our results suggested that dogs that exercise more than 3 h were more fearful towards thunder than dogs that exercise 1–3 h daily. Thus, more research is needed to investigate what amount of exercise is beneficial for protecting dogs against non-social fear. Recent studies have also revealed increased levels of stress-related biomarkers in fearful dogs^38,39^. On the other hand, earlier studies have shown that fearful dogs have difficulty in solving cognitive tasks and show more stress-related behaviour when solving the puzzle^57,58^, suggesting that fearful dogs are not ideal partners for training.

Dogs poorly socialised in their early life were more fearful. The early life until 12–14 weeks of age is a crucial phase that modifies a dog’s behaviour^28,59,60^. In Finland, puppies are usually rehomed at the age of 7 or 8 weeks, which leaves considerable socialisation rehearsal to do for the new owners. In our study, socialisation was important for avoiding noise sensitivity and fear of novel situations. Similar results for socialisation in noise sensitivity were observed previously^34^. To our knowledge, studies focused only on fear of novel situations and socialisation have not been performed. Thus, our study underlines the importance of socialisation in avoiding non-social fear.

We observed that dogs belonging to inexperienced dog owners had a higher likelihood of fear of fireworks, consistent with previous studies^34,61^. The owner’s dog experience was also associated with fear of surfaces and heights as the owner’s first dog had a higher likelihood of fear of surfaces and heights. We also found that dogs living in a larger family were more fearful towards novel situations. The same phenomenon was observed in general fearfulness in a previous study, which revealed that dogs living with several adults were more fearful than those living in families with only one adult, but fearfulness was not associated with the number of children^34^. Owners living in large families may not be able to pay as much attention to their dogs as single owners. However, the explanation for this association is uncertain and more research is needed.

We found that the presence of other dogs in the family was associated with noise sensitivity-related traits and fear of surfaces and heights. Only dogs had a higher likelihood of fear of fireworks, thunder, and surfaces and heights than dogs living with conspecifics. This result is consistent with a previous study, in which noise sensitivity was more common in dogs living without conspecifics^34^. The company of other dogs may thus reduce stress. An earlier study reported that dogs living with other dogs had a smaller physiological fear response to a simulated thunderstorm than dogs living without other dogs^62^.

We made the novel observation that dogs living in urban areas were more likely to show non-social fear than dogs living in rural areas. Studies on living environment (urban–rural axis) and non-social fear are limited. Blackwell and others found no evidence that the dog’s current living environment is associated with noise sensitivity^41^. A recent study revealed a similar association between living environment and social fear^36^. The authors suggested that urban environments are hectic and the populations of dogs and humans are denser. This can lead to increased stress in dogs which in turn can be expressed as a fearful behaviour. In contrast, the study also pointed out that in rural environments, dogs may not encounter fearful situations as often as urban dogs.

The questionnaire approach has some limitations, as questionnaires are somewhat subjective. However, many previous studies have revealed that behaviour questionnaires to have good reliability^30,63–66^. As we used an online questionnaire, dog owners that use social media were more likely to answer the questionnaire.

In conclusion, our results indicate that non-social fear in dogs is associated with multiple demographic and environmental factors. Genetics also play a role and this suggests that molecular studies in high-risk breeds are warranted. To avoid non-social fear, particular attention should be paid to early-life socialisation and an active and social life style. Given the rapid urbanisation of countries, further research should be performed to better understand the urban stressors to develop improved management approaches for dogs.

## Methods

### Questionnaire

We designed an online owner-completed questionnaire and collected information on dog behaviour from the owners of Finnish pet dogs. The questionnaire included an extensive background section, that considered, for example, the dog’s age, sex, breed, current number of other dogs in the household, hobbies, and puppyhood socialisation. Behaviour sections focused on the following seven canine anxiety traits: fear, aggression, noise sensitivity, fear of heights/surfaces, hyperactivity/inattention, separation anxiety, and compulsion. Here, we focused on non-social fear, specifically fear of surfaces and heights, noise sensitivity, and fear of novel situations. More specific categorisations and the questionnaire can be found as Supplementary material in the article by Salonen et al.^40^. We advertised the questionnaire on Facebook and via dog breed organisations.

### Noise sensitivity

The respondents were asked whether their dog showed fear of thunder or fireworks. If the dog showed fear, the owners were asked to estimate the frequency of symptoms (rarely, 0–20% of the time; sometimes, 20–40%; often, 40–60%; almost always, 60–100%; or always, 100% of the time). Dogs that showed fear of these sounds at least often (40–60% of the time) were placed in the high group and dogs that never showed fear were placed in the low group.

### Fear of novel situations

The respondents were asked whether their dog showed fear in new situations and to describe how often their dog showed fear (rarely, 0–20% of the time; sometimes, 20–40%; often, 40–60%; almost always, 60–100%; or always, 100% of the time). Dogs that showed fear in novel situations at least often (40–60% of the time) formed the high group and dogs that never showed fear formed the low group.

### Fear of surfaces and heights

The respondents were asked whether their dog had trouble walking in high places, including walking next to glass railings, walking over narrow bridges, climbing open riser stairs, climbing closed risers, and climbing metal stairs where you can see “through” the steps, and different surfaces, including walking on shiny floors, metal grids, and from one surface to another. The owners estimated how often their dog has trouble in these situations (never, 0; rarely, 1; sometimes, depends on the place, 2; often, 3; or always, 4). Dogs belonging to the low group did not show fear (0) towards any of these situations. Dogs belonging to the high group showed fear often (3) or always (4) in at least one situation.

### Demographic and environmental variables

We created new variables before analyses. Similar to article by Puurunen and others^36^, we only included breeds with adequate sample sizes (over 10 individuals both in low and high groups) in order to have enough individuals in high and low groups for logistic regression analysis. The criterion matched to the following 22 breeds and mixed breed dogs Border Collie, Cairn Terrier, Chihuahua (both coat types combined), Chinese Crested Dog (both coat types combined), Coton de Tuléar, Finnish Lapponian Dog, German Shepherd Dog, Golden Retriever, Irish Soft Coated Wheaten Terrier (later Wheaten Terrier), Jack Russell Terrier, Labrador Retriever, Lagotto Romagnolo, Lapponian Herder, Medium size Spitz, Miniature Poodle (Toy, Miniature, and Medium sizes), Miniature Schnauzer, Pembroke Welsh Corgi, Rough Collie, Shetland Sheepdog, Smooth Collie, Spanish Water Dog, and Staffordshire Bull Terrier (later Staff. Bull Terrier). Breeds with under 10 individuals in high and low groups were combined under the “other” breed group.

We created a new variable “body size” based on AKC and FCI breed standards. The dogs were categorised into small (height ≤ 35 cm), medium (height 36–49 cm), and large (height ≥ 50 cm) groups based on the breed’s average height. The body size of mixed breed dogs was unknown and thus mixed breed dogs were excluded when body size was included in analyses. The variable “socialisation” combined seven questions in which respondents answered how often the dog had met unfamiliar men, women, children or strange dogs, and how often the dog had visited a city centre or travelled by car or bus between the age of 7 weeks and 4 months (the critical socialisation period). All the questions had choices from never to several times a day (0, never; 1, 1–2 times during the puppyhood; 2, 1–2 times during the puppyhood to 2 times per month; 3, twice a month to twice a week; 4, twice a week to once a day; 5, several times a day). The scores were summed and thus the socialisation score varied between 0 and 35, with higher value indicating more socialisation events. We utilised a variable “family size” that had five categories (1, single, childless, living alone; 2, childless couple; 3, one-child family with one to two adults; 4, two-children family with one to two adults; 5, bigger family, more than two children or more than two adults). We included a variable describing the presence of conspecifics (“other dogs in the family”); either the dog was an only dog or the owner had other dogs as well. We also included the owner’s dog experience as a binomial variable (“owner’s dog experience”); either the dog was the owner’s first dog or not. Furthermore, we included the amount of exercise and participation in activities in the analyses. Exercise (“daily exercise”) consisted of active movement chosen by the owner (not including spending time alone in the yard) and was categorised into four categories (1, less than 60 min per day; 2, 1–2 h per day; 3, 2–3 h per day; and 4, over 3 h per day). The frequency of activities and training such as agility, obedience training, and hunting (“activities/training”) was categorised into three categories (1, never/seldom [once a year]; 2, sometimes [once a month/couple of times in a year]; and 3, at least weekly). Furthermore, we used the trait “fearfulness” as an explanatory variable. This variable categorised dogs into low, moderate, and high groups in general fearfulness. This was derived from the questionnaire section fear and included “fear of dogs”, “fear of strangers”, and “fear of novel situations”. A detailed categorisation for the “fearfulness” trait can be found in the article by Salonen and others^40^.

To examine the dog’s living environment outside of the home, we quantified the environmental land-use around the current home of the dog using the same method as in the article by Puurunen and others^36^. Geographical coordinates for each home were derived using addresses provided by the dog owners. The coverage of three land-use types (artificial surfaces, agricultural areas, and forest and semi-natural areas) were calculated within a 3-km buffer around the homes, using the publicly available land-use database CORINE2012 with a 25-m resolution. We simplified this information further into one continuous variable through a principal component analysis that essentially reduced the land-use information into a single rural–urban gradient. A higher “urban environment score” indicated a larger proportion of built environment.

Other variables used in the analyses were age, sex, and sterilisation status. All the behavioural, demographic, and environmental variables derived from the questionnaire data and included in the analyses are described in detail in Supplementary Table S6.

### Statistical analyses

We used logistic regression to analyse the effects of demographic and environmental factors on non-social fear, including fear of fireworks, fear of thunder, fear of surfaces and heights, and fear of novel situations. Initial data included 13,715 dogs. We excluded individuals with incomplete or missing responses. Thus, we obtained data of 5,822 dogs in fear of fireworks, 5,999 dogs in fear of thunder, 5,944 in fear of novel situations, and 2,594 in fear of heights and surfaces. We used these four traits as binary response variables in the analyses, fearful dogs as the event, and non-fearful dogs as the non-event.

We selected the explanatory variables based on previous studies. We used a forward stepwise model selection by Akaike Information Criterion (AIC) values. As there are behavioural differences in age and sex^40^, we made an initial model that included sex and age as explanatory variables. The whole AIC model selection process and final models are presented in the Supplementary Table S7. We tested for the interaction between sex and sterilisation for all models, and for the fear of novel situations model the interaction fitted better and was added in the final model. After we had chosen final models, we created datasets including only those variables that were selected for the final model to maximise sample sizes. Thus, final datasets included 9,613 dogs in fear of fireworks, 9,513 dogs in fear of thunder, 6,945 in fear of novel situations, and 2,932 in fear of heights and surfaces.

All statistical analyses were conducted using R version 3.6.1^67^. We checked the linearity assumption by fitting a generalised additive model with the package “gam”^68^. If the assumption was not met, we included both linear and quadratic variables in the model (e.g. age and age^2^). We inspected possible outliers in the datasets with the packages “broom”^69^ and “dplyr”^70^ and by plotting standardized residuals with the package “ggplot2”^71^. We tested the multicollinearity by requesting the variance inflation factor (VIF) with the package “car”^72^. We estimated the predictive ability of the models by calculating the area under the receiver operator characteristic curve (AUC) with the package “pROC”^73^ and AUCs were adequate. We calculated the estimated marginal means for categorical explanatory variables with the package “emmeans”^74^. We obtained the effects of continuous explanatory variables with the package “effects”^75^. We used analysis of variance (ANOVA) to obtain the overall effects of the explanatory variables with the package “car”^72^.

### Contrasts

We formed several a priori contrasts based on previous studies. In noise sensitivity-related subtraits (fear of fireworks and fear of thunder), we hypothesised that older dogs would be more fearful than younger dogs^41^, that female dogs would be more fearful than males^15^, and that dogs belonging to first-time dog owners (less experienced) would show more fearful behaviour^34^. The hypotheses and contrasts were the same in both fear of fireworks and fear of thunder analyses, except for the owner’s dog experience, which was included only in the fear of fireworks model. In fear of novel situations, we hypothesised that female dogs would be more fearful than male dogs^15,31^, and that younger dogs would be more fearful than older dogs^15,31,45^. In fear of surfaces and heights, we hypothesised that female dogs would show more fear of surfaces and heights than male dogs^15,31^, that smaller dogs would be more fearful than larger dogs^48,49^, and that the fearful dogs would also be fearful towards surfaces and heights.

We examined a priori contrasts and all pairwise comparisons between different levels of the included categorical variables with the package “emmeans”^74^. As the number of pairwise comparisons was high due to a large number of categorical variables, we corrected all p-values for false discovery rate (FDR) to decrease the probability of type I error. As contrasts between the different groups described above were chosen a priori, the p-values of these contrasts were not adjusted. We set the significance cut-off to p-value < 0.05.

### Ethics statement

The data were collected before the onset of the GDPR regulation according to the Finnish legislation https://www.finlex.fi/fi/laki/ajantasa/1999/19990523. This survey study focused on dogs and not human participants (dog owners) and therefore a specific ethical approval was not needed. We collected only names and addresses from the study participants (dog owners). Owners were informed that the participation is voluntary, confidential, and that the data are used only for scientific purposes. We received informed consent from all participants.

## Supplementary information

Supplementary Information 1.

Supplementary Information 2.

## Data Availability

The anonymised data is available as Supplementary material in the article by Salonen et al*.*^40^.
