# Supplementary material for: Active and social life is associated with lower non-social fearfulness in pet dogs
Source: Sci Rep. 2020 Aug 13;10:13774. doi: 10.1038/s41598-020-70722-7 (PMC7426946; doi:10.1038/s41598-020-70722-7)
Supplement: Supplementary file 1 — Supplementary Information 1. [file 41598_2020_70722_MOESM1_ESM.docx]

**Active and social life is associated with lower non-social fearfulness in pet dogs**

Hakanen, E.^1,2^, Mikkola, S.^1,2^, Salonen, M.^1,2^, Puurunen, J.^1,2^, Sulkama, S.^1,2^, Araujo, C.^1,2^, Lohi, H.*^1,2^

^1^Department of Veterinary Biosciences and Department of Medical and Clinical Genetics, University of Helsinki, Helsinki, Finland

^2^Folkhälsan Research Center, Helsinki, Finland

* Corresponding author:

Hannes Lohi, PhD, Professor

Email: [hannes.lohi@helsinki.fi](mailto:hannes.lohi@helsinki.fi) (HL)

**Supplementary information**

**Supplementary Table S1**. Descriptive statistics. n= 9,613 (fear of fireworks), n = 9,513 (fear of thunder), n = 6,945 (fear of novel situations), N = 2,932 (fear of heights and surfaces).

**Fear of fireworks**

|  |  | mean | sd |
| --- | --- | --- | --- |
| Age (years) |  | 4.78 | 3.21 |
| Socialisation score |  | 21.78 | 4.36 |
|  |  | N | % |
| Sex | Female | 4992 | 51.93 |
|  | Male | 4621 | 48.07 |
| Sterilisation | Intact | 6830 | 71.05 |
|  | Neutered | 2783 | 28.95 |
| Breed | Border Collie | 203 | 2.11 |
|  | Cairn Terrier | 71 | 0.74 |
|  | Chihuahua | 79 | 0.82 |
|  | Chinese Crested Dog | 101 | 1.05 |
|  | Coton de Tulear | 82 | 0.85 |
|  | Finnish Lapponian Dog | 386 | 4.02 |
|  | German Shepherd Dog | 331 | 3.44 |
|  | Golden Retriever | 138 | 1.44 |
|  | Irish Soft Coated Wheaten Terrier | 218 | 2.50 |
|  | Jack Russell Terrier | 112 | 1.17 |
|  | Labrador Retriever | 361 | 3.76 |
|  | Lagotto Romagnolo | 170 | 1.77 |
|  | Lapponian Herder | 201 | 2.09 |
|  | Medium size Spitz | 89 | 0.93 |
|  | Miniature Poodle | 218 | 2.27 |
|  | Miniature Schnauzer | 194 | 2.02 |
|  | Mixed Breed | 214 | 2.23 |
|  | Other | 5384 | 56.01 |
|  | Pembroke Welsh Corgi | 72 | 0.75 |
|  | Rough Collie | 182 | 1.89 |
|  | Shetland Sheepdog | 303 | 3.15 |
|  | Smooth Collie | 143 | 1.49 |
|  | Spanish Water Dog | 192 | 2.00 |
|  | Staffordshire Bull Terrier | 147 | 1.53 |
| Activities/training | Never/seldom | 2391 | 24.87 |
|  | Sometimes | 2719 | 28.28 |
|  | At least weekly | 4503 | 46.84 |
| Dogs in family | Only dog in family | 3461 | 36.0 |
|  | Other dogs in family | 6152 | 64.0 |
| Owner’s dog experience | The dog is first dog | 2688 | 27.96 |
|  | The dog is not first dog | 6925 | 72.04 |

**Fear of thunder**

|  |  | mean | sd |
| --- | --- | --- | --- |
| Age (years) |  | 4.72 | 3.20 |
| Socialisation score |  | 21.79 | 4.33 |
|  |  | N | % |
| Sex | Female | 4926 | 51.78 |
|  | Male | 4587 | 48.21 |
| Sterilisation | Intact | 6848 | 71.99 |
|  | Neutered | 2665 | 28.01 |
| Breed | Border Collie | 224 | 2.35 |
|  | Cairn Terrier | 71 | 0.75 |
|  | Chihuahua | 87 | 0.91 |
|  | Chinese Crested Dog | 106 | 1.11 |
|  | Coton de Tulear | 82 | 0.86 |
|  | Finnish Lapponian Dog | 369 | 3.88 |
|  | German Shepherd Dog | 350 | 3.68 |
|  | Golden Retriever | 137 | 1.44 |
|  | Irish Soft Coated Wheaten Terrier | 258 | 2.71 |
|  | Jack Russell Terrier | 118 | 1.24 |
|  | Labrador Retriever | 363 | 3.82 |
|  | Lagotto Romagnolo | 179 | 1.88 |
|  | Lapponian Herder | 216 | 2.27 |
|  | Medium size Spitz | 95 | 1.00 |
|  | Miniature Poodle | 207 | 2.18 |
|  | Miniature Schnauzer | 188 | 1.98 |
|  | Other | 5406 | 56.83 |
|  | Pembroke Welsh Corgi | 72 | 0.76 |
|  | Rough Collie | 189 | 1.99 |
|  | Shetland Sheepdog | 298 | 3.13 |
|  | Smooth Collie | 145 | 1.52 |
|  | Spanish Water Dog | 196 | 2.06 |
|  | Staffordshire Bull Terrier | 157 | 1.65 |
| Activities/training | Never/seldom | 2253 | 23.68 |
|  | Sometimes | 2708 | 28.47 |
|  | At least weekly | 4552 | 47.85 |
| Dogs in family | Only dog in family | 3355 | 35.26 |
|  | Other dogs in family | 6158 | 64.73 |
| Daily exercise | Less than 1 hour | 721 | 7.58 |
|  | 1-2 hours | 3908 | 41.08 |
|  | 2-3 hours | 3666 | 38.54 |
|  | More than 3 hours | 1218 | 12.80 |
| Body size | Small | 2272 | 23.88 |
|  | Medium | 3274 | 34.42 |
|  | Large | 3967 | 41.70 |

**Fear of novel situations**

|  |  | mean | sd |
| --- | --- | --- | --- |
| Age (years) |  | 4.63 | 3.28 |
| Socialisation score |  | 21.90 | 4.32 |
| Urban environment score |  | -0.03 | 1.39 |
|  |  | N | % |
| Sex | Female | 3633 | 52.31 |
|  | Male | 3312 | 47.69 |
| Sterilisation | Intact | 5127 | 73.82 |
|  | Neutered | 1818 | 26.18 |
| Sex*sterilisation | Intact female | 2620 | 37.72 |
|  | Intact male | 2507 | 36.10 |
|  | Neutered female | 1013 | 14.59 |
|  | Neutered male | 805 | 11.59 |
| Family size | Single | 1423 | 20.49 |
|  | Couple | 3004 | 43.25 |
|  | Family with one or two adults and one child | 805 | 11.59 |
|  | Family with one or two adults and two children | 853 | 12.28 |
|  | Family with three or more adults and/or three or more children | 860 | 12.38 |
| Activities/training | Never/seldom | 1654 | 23.82 |
|  | Sometimes | 1998 | 28.77 |
|  | At least weekly | 3293 | 47.42 |

**Fear of surfaces and heights**

|  |  | mean | sd |
| --- | --- | --- | --- |
| Age (years) |  | 5.05 | 3.24 |
| Socialisation score |  | 21.99 | 4.57 |
| Urban environment score |  | 0.09 | 1.39 |
|  |  | N | % |
| Sex | Female | 1530 | 52.18 |
|  | Male | 1402 | 47.82 |
| Breed | Border Collie | 84 | 2.86 |
|  | Cairn Terrier | 24 | 0.82 |
|  | Chihuahua | 32 | 1.09 |
|  | Chinese Crested Dog | 29 | 0.99 |
|  | Coton de Tulear | 42 | 1.43 |
|  | Finnish Lapponian Dog | 99 | 3.38 |
|  | German Shepherd Dog | 116 | 3.96 |
|  | Golden Retriever | 40 | 1.36 |
|  | Irish Soft Coated Wheaten Terrier | 71 | 2.42 |
|  | Jack Russell Terrier | 40 | 1.36 |
|  | Labrador Retriever | 112 | 3.82 |
|  | Lagotto Romagnolo | 63 | 2.15 |
|  | Lapponian Herder | 48 | 1.64 |
|  | Medium size Spitz | 28 | 0.95 |
|  | Miniature Poodle | 71 | 2.59 |
|  | Miniature Schnauzer | 60 | 2.05 |
|  | Other | 1645 | 56.11 |
|  | Pembroke Welsh Corgi | 18 | 0.61 |
|  | Rough Collie | 77 | 2.63 |
|  | Shetland Sheepdog | 85 | 2.90 |
|  | Smooth Collie | 47 | 1.60 |
|  | Spanish Water Dog | 54 | 1.84 |
|  | Staffordshire Bull Terrier | 42 | 1.43 |
| Activities/training | Never/seldom | 767 | 26.16 |
|  | Sometimes | 809 | 27.59 |
|  | At least weekly | 1356 | 46.25 |
| Dogs in family | Only dog in family | 1017 | 34.69 |
|  | Other dogs in family | 1915 | 65.31 |
| Owner’s dog experience | The dog is first dog | 803 | 27.39 |
|  | The dog is not first dog | 2129 | 72.61 |
| Daily exercise | Less than 1 hour | 201 | 6.86 |
|  | 1-2 hours | 1186 | 40.45 |
|  | 2-3 hours | 1107 | 37.76 |
|  | More than 3 hours | 438 | 14.94 |
| Body size | Small | 788 | 26.88 |
|  | Medium | 912 | 31.11 |
|  | Large | 1232 | 42.02 |
| Fearfulness | Low | 1246 | 42.50 |
|  | Moderate | 766 | 26.13 |
|  | High | 920 | 31.38 |

**Supplementary Table S2.** Contrasts between levels of categorical variables in the fear of fireworks logistic regression analysis.

| **Variable** | **Contrasts** | **OR** | **Lower 95% Cl** | **Upper 95% Cl** | **p-value** |
| --- | --- | --- | --- | --- | --- |
| Sex | Male vs. female | 1.01 | 0.917 | 1.1 | 0.8908* |
| Owner’s dog experience | The first dog vs. not the first dog | 1.53 | 1.37 | 1.7 | **<0.0001*** |
| Sterilisation | Intact vs. neutered | 0.749 | 0.675 | 0.832 | **0.0004** |
| Activities/training | Never/seldom vs. sometimes | 1.26 | 1.112 | 1.43 | **0.0031** |
|  | Never/seldom vs. at least weekly | 1.41 | 1.251 | 1.58 | **0.0004** |
|  | Sometimes vs. at least weekly | 1.12 | 0.998 | 1.25 | 0.2123 |
| Dogs in the family | Only dog vs. other dogs | 1.44 | 1.3 | 1.6 | **0.0004** |
| P-values are controlled for false discovery rate except for *a priori* contrasts. A priori effects are denoted with*. Significant effects are in bold (p-value < 0.05). OR = odds ratio, Cl = confidence level, n = 9,613. | | | | | |

**Supplementary Table S3.** Contrasts between levels of categorical variables in the fear of thunder logistic regression analysis.

| **Variable** | **Contrasts** | **OR** | **Lower 95% Cl** | **Upper 95% Cl** | **p-value** |
| --- | --- | --- | --- | --- | --- |
| Sex | Male vs. female | 0.961 | 0.859 | 1.08 | 0.4863* |
| Sterilisation | Intact vs. neutered | 0.665 | 0.589 | 0.751 | **0.0006** |
| Dogs in the family | Only dog vs. other dogs | 1.45 | 1.28 | 1.63 | **0.0006** |
| Body size | Large vs. small | 0.72 | 0.601 | 0.863 | **0.0004*** |
|  | Small vs. medium | 0.818 | 0.673 | 0.995 | 0.0981 |
|  | Medium vs. large | 0.880 | 0.733 | 1.06 | 0.1669 |
| Activities/training | Never/seldom vs. sometimes | 1.11 | 0.961 | 1.29 | 0.1522 |
|  | Never/seldom vs. at least weekly | 1.36 | 1.18 | 1.58 | **0.0001** |
|  | Sometimes vs. at least weekly | 1.23 | 1.07 | 1.41 | **0.0037** |
| Daily exercise | Less than 1 hour vs. 1-2 hours | 1.02 | 0.829 | 1.25 | 0.8641 |
|  | Less than 1 hour vs. 2-3 hours | 1.08 | 0.874 | 1.34 | 0.4705 |
|  | Less than 1 hour vs. more than 3 hours | 0.844 | 0.658 | 1.08 | 0.1792 |
|  | 1-2 hours vs. 2-3 hours | 1.06 | 0.935 | 1.21 | 0.353 |
|  | 1-2 hours vs. more than 3 hours | 0.829 | 0.692 | 0.993 | **0.0412** |
|  | 2-3 hours vs. more than 3 hours | 0.78 | 0.649 | 0.937 | **0.008** |
| P-values are controlled for false discovery rate except for *a priori* contrasts. A priori effects are denoted with*. Significant effects are in bold (p-value < 0.05). OR = odds ratio, Cl = confidence level, n = 9,513. | | | | | |

**Supplementary Table S4.** Contrasts between levels of categorical variables in the “fear of novel situations” logistic regression analysis.

| **Variable** | **Contrasts** | **OR** | **Lower 95% Cl** | **Upper 95% Cl** | **p-value** |
| --- | --- | --- | --- | --- | --- |
| Sex | Male vs. female | 0.999 | 0.733 | 1.36 | 0.9942* |
| Sterilisation | Intact vs. neutered | 0.368 | 0.263 | 0.515 | **0.0003** |
| Sex*sterilisation | Intact male vs. intact female | 0.813 | 0.679 | 0.973 | **0.0373** |
|  | Intact male vs. neutered male | 0.494 | 0.392 | 0.621 | **0.0003** |
|  | Intact male vs. neutered female | 0.607 | 0.481 | 0.765 | **0.0003** |
|  | Intact female vs. neutered male | 0.607 | 0.406 | 0.759 | **0.0003** |
|  | Intact female vs. neutered female | 0.746 | 0.595 | 0.936 | **0.0187** |
|  | Neutered male vs. neutered female | 1.229 | 0.955 | 1.58 | 0.1292 |
| Activities/training | Never/seldom vs. sometimes | 1.67 | 1.381 | 2.02 | **0.0003** |
|  | Never/seldom vs. at least weekly | 1.84 | 1.54 | 2.2 | **0.0003** |
|  | Sometimes vs. at least weekly | 1.1 | 0.918 | 1.32 | 0.3159 |
| Family | Single vs. couple | 0.815 | 0.663 | 1.002 | 0.0718 |
|  | Single vs. one child | 0.772 | 0.585 | 1.019 | 0.0887 |
|  | Single vs. two children | 0.661 | 0.506 | 0.865 | **0.0048** |
|  | Single vs. larger family | 0.567 | 0.438 | 0.734 | **0.0003** |
|  | Couple vs. one child | 0.947 | 0.743 | 1.207 | 0.6602 |
|  | Couple vs. two children | 0.812 | 0.645 | 1.022 | 0.0951 |
|  | Couple vs. larger family | 0.695 | 0.558 | 0.866 | **0.0025** |
|  | One child vs. two children | 0.857 | 0.639 | 1.15 | 0.3159 |
|  | One child vs. larger family | 0.734 | 0.552 | 0.977 | 0.0504 |
|  | Two children vs. larger family | 0.857 | 0.65 | 1.129 | 0.3091 |
| P-values are controlled for false discovery rate except for *a priori* contrasts. *A priori* effects are denoted with*. Significant effects are in bold (p-value < 0.05). OR = odds ratio, Cl = confidence level, n = 6,945. | | | | | |

**Supplementary Table S5.** Contrasts between levels of categorical variables in the fear of surfaces and heights logistic regression analysis.

| **Variable** | **Contrasts** | **OR** | **Lower 95% CL** | **Upper 95% CL** | **p-value** | |
| --- | --- | --- | --- | --- | --- | --- |
| Sex | Male vs. female | 1.01 | 0.854 | 1.19 | 0.9111* | |
| Body size | Large vs. medium | 1.079 | 0.832 | 1.399 | 0.7931 | |
|  | Large vs. small | 0.398 | 0.303 | 0.523 | **<0.0001*** | |
|  | Medium vs. small | 0.369 | 0.274 | 0.497 | **0.0021** | |
| Dogs in the family | Only dog vs. other dogs | 1.88 | 1.55 | 2.28 | **0.0021** | |
| Fearfulness | Low vs. moderate | 0.61 | 0.499 | 0.746 | **<0.0001*** | |
|  | Low vs. high | 0.359 | 0.291 | 0.441 | **<0.0001*** | |
|  | Moderate vs. high | 0.588 | 0.468 | 0.738 | **<0.0001*** | |
| Owner’s dog experience | The first dog vs. not first dog | 1.49 | 1.21 | 1.82 | **0.0021** | |
| Daily exercise | Less than 1 hour vs. 1-2 hours | 1.27 | 0.876 | 1.85 | 0.3919 | |
|  | Less than 1 hour vs. 2-3 hours | 1.54 | 1.057 | 2.25 | 0.1216 | |
|  | Less than 1 hour vs. more than 3 hours | 1.74 | 1.149 | 2.62 | 0.0632 | |
|  | 1-2 hours vs. 2-3 hours | 1.21 | 1.002 | 1.46 | 0.2045 | |
|  | 1-2 hours vs. more than 3 hours | 1.36 | 1.058 | 1.76 | 0.0928 | |
|  | 2-3 hours vs. more than 3 hours | 1.13 | 0.877 | 1.45 | 0.5851 | |
| Activities/training | Never/seldom vs. sometimes | 2.06 | 1.612 | 2.63 | **0.0021** | |
|  | Never/seldom vs. at least weekly | 2.26 | 1.793 | 2.85 | **0.0021** | |
|  | Sometimes vs. at least weekly | 1.1 | 0.903 | 1.34 | 0.5851 | |
| P-values are controlled for false discovery rate except for *a priori* contrasts. *A priori* effects are denoted with*. Significant effects are in bold (p-value < 0.05). OR = odds ratio, Cl = confidence level, n = 2,932. | | | | | |  |

**Supplementary Table S6.** Demographic and environmental variables derived from the owner-filled online canine behavioural questionnaire.

| **Variable** | **Description** | **Possible values** |
| --- | --- | --- |
| Fear of thunder | Binomial (event/non-event) variable. Fearful dogs showed fear of thunder at least often (40-60% of the times) (event). Non-fearful dogs never showed fear of thunder (non-event). | 1: high group  0: low group |
| Fear of fireworks | Binomial (event/non-event) variable. Fearful dogs showed fear of fireworks at least often (40-60% of the times) (event). Non-fearful dogs never showed fear of fireworks (non-event). | 1: high group  0: low group |
| Fear of novel situations | Binomial (event/non-event) variable. Fearful dogs were reported to show fearful behaviour often (40-60% of the occasions) (event). Non-fearful dogs did not show fear towards new situations and the owners did not report a prevalence for fear (non-event). | 1: high group  0: low group |
| Fear of surfaces and heights | Binomial (event/non-event) variable. Dogs belonging to the high group showed fear of surfaces and heights at least often (event). Dogs belonging to low group did not show fear towards to these situations (non-event). | 1: high group  0: low group |
| Breed | Categorial variable. Owners reported the breed of their dog. We chose 22 breeds that had the most adequate sample size. Breeds with fewer respondents were combined under ‘other’ breed group. Mixed breed was also added in data.  Notes for specific breeds:  For breed, for Chihuahua both coat types were combined  For breed, for Chinese Crested Dog both coat types were combined  For breed, for Poodle Toy, Miniature, and Medium sizes were combined | Border Collie, Cairn Terrier, Chihuahua, Chinese Crested Dog, Coton de Tuléar, Finnish Lapponian Dog, German Shepherd Dog, Golden Retriever, Irish Soft Coated Wheaten Terrier, Jack Russell Terrier, Labrador Retriever, Lagotto Romagnolo, Lapponian Herder, Medium size Spitz, Miniature Poodle. Miniature Schnauzer, Mixed breed, Pembroke Welsh Corgi, Rough Collie, Shetland Sheepdog, Smooth Collie, Spanish Water Dog, and Staffordshire Bull Terrier |
| Age (years) | Continuous variable. The age of dog when owner filled the behavioural questionnaire. | 0.2 – 17.9 |
| Sterilisation | Binomial variable. Owners reported if their dog was neutered or not. | 0: intact  1: neutered |
| Sex*sterilisation interaction | Categorical variable, which combined the sterilisation (intact or neutered) and dog’s sex. | intact male, neutered male, intact female, neutered female |
| Socialisation score | Continuous variable. A sum of the frequencies of how often (0, never; 1, 1 to 2 times during the puppyhood; 2, 1 to 2 times during the puppyhood to 2 times per month; 3, twice a month to twice a week; 4, twice a week to once a day; 5, several times a day) the dog met unfamiliar men, women, and children, unfamiliar adult dogs, visited city or other place with traffic and many people, and travelled by car and bus, when the dog was 7-16 weeks old. | 0 – 35 |
| Urban environment score | Continuous variable. Describes the environmental land-use around the dog’s home. The geographical coordinates for each home were derived from the addresses provided by the owners. The coverage of three land-use types (artificial surfaces, agricultural areas, forests and semi-natural areas) were calculated using CORINE2012 within a 3-kilometre range around the homes. Through PCA, the coverages were simplified into one continuous variable, where higher scores indicate a more urban environment. | -2.28 – 3.11 |
| Activities/training | Categorial variable. Describes how often the dog participated in activities or training. | 1, never/seldom; 2, sometimes; 3, at least weekly |
| Daily exercise | Categorial variable. Describes the amount of dog’s daily exercise in hours. | 1, < 1 hour; 2, 1-2 hours; 3, 2-3 hours; 4, > 3 hours |
| Owner’s dog experience | Binomial variable. Describes the owner’s experience with dogs. | 1, the dog was the owner’s first dog; 2, the dog was not the owner’s first dog |
| Dogs in the family | Binomial variable. Describes the number of dogs in the family. | 1, the dog was the only dog in the family; 2, there was also other dogs in the family |
| Family size | Categorial variable. Describes the size of the family where the dog lived. | 1, single; 2, couple; 3, family with one or two adults and one child; 4, family with one or two adults and two children; 5, family with three or more adults and/or three or more children |
| Weaning age | Categorial variable. The weaning ages were divided into four categories: | 1, < 7 weeks of age; 2, at 7 weeks of age; 3, at 8 weeks of age; 4, > 8 weeks of age |
| Daily time spent alone | Categorial variable. Describes the daily time that the dog spent alone at home without the presence of people. | 1, < 3 hours; 2, 3-6 hours; 3, 6-8 hours; 4, > 8 hours |
| Body size | Categorial variable. Dogs were divided into three different body size categories, small, medium and large, based on the average height of the breed. | Small, ≤ 35 cm; medium, 36-49 cm; large, ≥ 50 cm |
| Fearfulness | Categorial variable. The fear section of the questionnaire consisted of three fear subtraits: fear of strangers, other dogs, and novel situations. The dogs that showed fear at least often (40-60% of the times) constituted the high group and dogs that never showed fear constituted the low group in all subtraits. Furthermore, low fear dogs never barked or growled at strangers or other dogs. | 0: low group  1: moderate group  2: high group |

**Supplementary Table S7**. The AIC model selection and the final models of logistic regression analyses. n = 5,822 (fear of fireworks), N = 5,999 (fear of thunder), n = 5,944 (fear of novel situations), n = 2,594 (fear of heights and surfaces).

| **Fear of fireworks** | | | | | | | | |  |
| --- | --- | --- | --- | --- | --- | --- | --- | --- | --- |
| Model | AIC | | Breed added | Owner’s dog experience added | Activities  /training added | Sterilisation added | Socialization  score added | Dogs in the family added | |
| Basic model (sex and age) | 6963.8 |  | |  |  |  |  |  | |
| Breed | **6849.5** | | 6849.5 |  |  |  |  |  | |
| Owner’s dog experience | 6893.7 | | **6798.5** | 6798.5 |  |  |  |  | |
| Activities/training | 6906.3 | | 6802.4 | **6758.5** | 6758.5 |  |  |  | |
| Sterilisation | 6930.6 | | 6814.5 | 6769.7 | **6734.4** | 6734.5 |  |  | |
| Socialisation score | 6946.3 | | 6828.7 | 6772.4 | 6736.8 | **6713.6** | 6713.6 |  | |
| Dogs in the family | 6912.3 | | 6802.1 | 6772.7 | 6742.1 | 6718.3 | **6694.9** | **6694.9** | |
| Body size | 6929.4 | | 6845.4 | 6798.0 | 6759.7 | 6736.0 | 6714.6 | 6696.3 | |
| Weaning age | 6963.3 | | 6849.0 | 6797.9 | 6759.8 | 6735.9 | 6716.1 | 6698.2 | |
| Daily exercise | 6962.3 | | 6847.8 | 6799.2 | 6762.1 | 6738.2 | 6718.8 | 6700.1 | |
| Urban environment score | 6961.1 | | 6849.1 | 6800.4 | 6760.4 | 6736.4 | 6715.2 | 6696.8 | |
| Daily time spent alone | 6946.3 | | 6851.0 | 6800.2 | 6760.0 | 6735.4 | 6713.3 | 6695.2 | |
| *The interaction term between sex and sterilisation was tested but not included in the final model as it did not improve the AIC value of the model.*  Sex*sterilisation | | | | | | | | 6696.7 | |
| Final model: age + sex + breed + owner’s dog experience + activities/training + sterilisation + socialisation score + dog in the family | | | | | | | | | |

| **Fear of thunder** | | | | | | | | | |
| --- | --- | --- | --- | --- | --- | --- | --- | --- | --- |
| Model | AIC | | Breed added | Sterilisation added | Activities/ training added | Socialisation score  added | Dogs in the family added | Body size  added | Daily exercise added |
| Basic model (sex and age) | 5441.5 |  | |  |  |  |  |  |  |
| Breed | **5360.1** | | 5360.1 |  |  |  |  |  |  |
| Sterilisation | 5410.4 | | **5329.0** | 5329.0 |  |  |  |  |  |
| Activities/training | 5412.4 | | 5336.1 | **5308.8** | 5308.8 |  |  |  |  |
| Socialisation score | 5423.4 | | 5340.2 | 5309.7 | **5292.5** | 5292.5 |  |  |  |
| Dogs in the family | 5419.6 | | 5340.2 | 5311.2 | 5297.2 | **5277.7** | 5277.7 |  |  |
| Body size | 5422.4 | | 5353.8 | 5322.7 | 5306.5 | 5289.5 | **5275.2** | 5275.2 |  |
| Daily exercise | 5436.2 | | 5353.7 | 5322.7 | 5306.4 | 5291.7 | 5275.9 | **5272.8** | **5272.8** |
| Weaning age | 5437.1 | | 5354.3 | 5323.3 | 5304.9 | 5290.2 | 5276.4 | 5274.2 | 5272.4 |
| Urban environment score | 5443.5 | | 5361.7 | 5330.0 | 5309.2 | 5294.3 | 5277.9 | 5274.4 | 5272.4 |
| Owner’s dog experience | 5429.8 | | 5354.9 | 5326.0 | 5307.0 | 5289.3 | 5277.8 | 5275.9 | 5273.4 |
| Daily time spent alone | 5439.5 | | 5359.3 | 5326.9 | 5308.4 | 5291.5 | 6277.4 | 5274.9 | 5272.9 |
| *The interaction term between sex and sterilisation was tested but not included in the final model as it did not improve the AIC value of the model.*  Sex*sterilisation | | | | | | | | | 5273.7 |
| Final model: age + sex + breed + sterilisation + activities/training + socialisation score + dogs in the family + body size + daily exercise | | | | | | | | | |

| **Fear of novel situations** | | | | | | |
| --- | --- | --- | --- | --- | --- | --- |
| Model | AIC | Socialisation score added | Activities/  training added | Sterilisation added | Urban environment score added | Family size added |
| Basic model (sex and age) | 4389.4 |  |  |  |  |  |
| Socialisation score | **4260.1** | 4260.1 |  |  |  |  |
| Activities/training | 4331.7 | **4213.4** | 4213.4 |  |  |  |
| Sterilisation | 4352.4 | 4225.0 | **4184.7** | 4184.7 |  |  |
| Urban environment score | 4388.0 | 4244.0 | 4203.1 | **4176.2** | 4176.2 |  |
| Family size | 4387.2 | 4255.5 | 4209.9 | 4179.3 | **4167.5** | **4167.5** |
| Body size | 4390.9 | 4263.0 | 4214.9 | 4186.0 | 4175.4 | 4166.4 |
| Daily exercise | 4376.5 | 4256.5 | 4213.4 | 4185.2 | 4177.0 | 4168.5 |
| Dogs in the family | 4388.4 | 4254.5 | 4212.8 | 4184.4 | 4177.6 | 4168.9 |
| Weaning age | 4385.4 | 4260.6 | 4215.1 | 4186.5 | 41782.2 | 4169.3 |
| Owner’s dog experience | 4391.4 | 4261.1 | 4215.2 | 4186.7 | 4177.9 | 4169.3 |
| Breed | 4396.5 | 4270.2 | 4224.0 | 4194.8 | 4185.6 | 4176.1 |
| Daily time spent alone | 4393.5 | 4264.6 | 4218.3 | 4189.3 | 4180.7 | 4172.6 |
| *The interaction term between sex and sterilisation was significant and improved the AIC value of the model, and thus the interaction term (sex*sterilisation) was included in the final model.*  Sex*sterilisation | | | | | | 4163.6 |
| Final model: age + sex + socialisation score + activities/training + sterilisation + urban environment score + family size + sex*sterilisation | | | | | | |

| **Fear of surfaces and heights** | | | | | | | | | | |
| --- | --- | --- | --- | --- | --- | --- | --- | --- | --- | --- |
| Model | AIC | Fear fulness  added | Body size added | Dogs in the family added | Activities/  training added | Breed added | Owner’s dog experience  added | Socialisation score added | Urban environment score added | Daily exercise added |
| Basic model (sex and age) | 3499.8 |  |  |  |  |  |  |  |  |  |
| Fearfulness | **3312.5** | 3312.5 |  |  |  |  |  |  |  |  |
| Body size | 3357.9 | **3203.0** | 3203.0 |  |  |  |  |  |  |  |
| Dogs in the family | 3402.5 | 3234.9 | **3127.3** | 3127.3 |  |  |  |  |  |  |
| Activities/training | 3345.2 | 3211.1 | 3134.3 | **3079.0** | 3079.0 |  |  |  |  |  |
| Breed | 3423.7 | 3250.9 | 3174.8 | 3102.7 | **3053.6** | 3053.6 |  |  |  |  |
| Owner’s dog experience | 3438.4 | 3265.2 | 3168.3 | 3113.0 | 3066.4 | **3042.4** | 3042.4 |  |  |  |
| Socialisation score | 3476.0 | 3305.5 | 3194.7 | 3115.9 | 3070.1 | 3044.0 | **3031.1** | 3031.1 |  |  |
| Urban environment score | 3445.6 | 3275.7 | 3181.0 | 3119.0 | 3071.2 | 3047.3 | 3037.9 | **3022.7** | 3022.7 |  |
| Daily exercise | 3458.7 | 3290.4 | 3191.3 | 3120.2 | 3074.6 | 3047.1 | 3035.7 | 3026.8 | **3019.8** | **3019.8** |
| Sterilisation | 3484.6 | 3308.1 | 3198.4 | 3123.4 | 3076.9 | 3051.3 | 3041.2 | 3030.5 | 3022.2 | 3019.6 |
| Weaning age | 3502.7 | 3316.6 | 3207.0 | 3130.4 | 3081.1 | 3057.2 | 3046.0 | 3034.0 | 3025.5 | 3022.7 |
| Family size | 3504.3 | 3315.5 | 3207.7 | 3131.2 | 3082.6 | 3057.6 | 3046.9 | 3035.8 | 3028.1 | 3024.4 |
| Daily time spent alone | 3504.3 | 3316.9 | 3207.0 | 3131.1 | 3082.4 | 3056.7 | 3045.8 | 3034.7 | 3026.9 | 3024.6 |
| *The interaction term between sex and sterilisation was tested but not included in the final model as it did not improve the AIC value of the model.*  Sex*sterilisation | | | | | | | | | | 3019.9 |
| Final model: age + sex + fearfulness + body size + dogs in the family + activities/training + breed + owner’s dog experience + socialisation score + urban environment score + daily exercise | | | | | | | | | | |

**Supplementary Figure S1.** Logistic regression analyses for the effects of sex, sterilisation, and owner’s dog experience in the fear of fireworks. **a.** There was no significant difference between sexes in fear of fireworks. **b.** Neutered dogs were more fearful towards the sound of fireworks than intact dogs. **c.** Owners’ first dogs were more likely to have fear of fireworks. Error bars indicate the 95% confidence limits. n = 9,613.

**Supplementary Figure S2.** Logistic regression analyses for the effects of sex, daily exercise, sterilisation, and body size in the fear of thunder. **a.** There was no significant difference between sexes in fear of thunder. **b.** The only significant differences were seen in dogs that exercised 1-2 hours or 2-3 hours were less fearful toward thunder than dogs that exercised more than 3 hours. **c.** Neutered dogs were more fearful towards the sound of thunder than intact dogs. **d.** Small dogs were more fearful towards thunder than medium and large dogs. Error bars indicate the 95% confidence limits. n = 9,513.

**Supplementary Figure S3.** Logistic regression analyses for **t**he effect of family size in the fear of novel situations. **a.** Dogs living in larger families had a higher likelihood to be afraid of novel situations. The dogs living with one adult (“single”) were less likely to have fear of novel situations than the dogs living in families with two children or a larger family. Dogs living with couples were less likely fearful than the dogs living in a larger family. Error bars indicate the 95% confidence limits. n = 6,945.

**Supplementary Figure S4.** Logistic regression analyses for the effects of age, socialisation, owner’s dog experience, sex, and daily exercise in the fear of surfaces and heights. **a.** Age had no significant effect on fear of surfaces and heights. **b.** There was no significant association between the socialisation score and fear of heights and surfaces. **c.** Owners’ first dogs were more likely to have fear of surfaces and heights. **d.** There was no significant difference between sexes in fear of surfaces and heights. **e.** The amount of exercise did not differ between fearful and non-fearful dogs. Grey lines (a, b) and error bars (c, d, e) indicate the 95% confidence limits. n = 2,932.
